# Longitudinal study of urban malaria in a cohort of Ugandan children: description of study site, census and recruitment

**DOI:** 10.1186/1475-2875-5-18

**Published:** 2006-03-21

**Authors:** Jennifer C Davis, Tamara D Clark, Sarah K Kemble, Nalugwa Talemwa, Denise Njama-Meya, Sarah G Staedke, Grant Dorsey

**Affiliations:** 1 Department of Medicine, San Francisco General Hospital, University of California, San Francisco, California, USA; 2 Makerere University Medical School, Kampala, Uganda

## Abstract

**Background:**

Studies of malaria in well-defined cohorts offer important data about the epidemiology of this complex disease, but few have been done in urban African populations. To generate a sampling frame for a longitudinal study of malaria incidence and treatment in Kampala, Uganda, a census, mapping and survey project was conducted.

**Methods:**

All households in a geographically defined area were enumerated and mapped. Probability sampling was used to recruit a representative sample of children and collect baseline descriptive data for future longitudinal studies.

**Results:**

16,172 residents living in 4931 households in a densely-populated community (18,824 persons/km^2^) were enumerated. A total of 582 households were approached with at least one child less than 10 years of age in order to recruit 601 children living in 322 households. At enrollment, 19% were parasitaemic, 24% were anaemic, 43% used bednets, and 6% used insecticide-treated nets. Low G6PD activity (OR = 0.33, P = 0.009) and bednet use (OR = 0.64, P = 0.045) were associated with a decreased risk of parasitaemia. Increasing age (OR = 0.62 for each year, P < 0.001) and bednet use (OR = 0.58, P = 0.02) were associated with a decreased risk of anaemia

**Conclusion:**

Detailed surveys of target populations in urban Africa can provide valuable descriptive data and provide a sampling frame for recruitment of representative cohorts for longitudinal studies. Plans to use a multi-disciplinary approach to improve the understanding of the distribution and determinants of malaria incidence and response to therapy in this population are discussed.

## Background

Malaria remains one of the most serious global health problems. There are an estimated one million deaths each year, with nearly 75% occurring in children living in sub-Saharan Africa [1]. Malaria control in resource poor countries has been severely affected by growing resistance to commonly used anti-malarial drugs and low utilization of preventative measures. New tools, such as highly effective artemisinin-based combination-therapy (ACT) and long-acting insecticide-treated nets (ITNs), offer great hope for Africa [2,3]. However, a better understanding of the epidemiology of malaria is needed to maximize the impact of these interventions.

Complex interactions between the host, parasite, and mosquito vector leads to wide variability in the risk of malaria and its clinical manifestations, ranging from asymptomatic parasitaemia to severe disease [4]. In highly endemic areas, malaria can cause repeated episodes of disease, especially in less immune younger children. This can lead to both short-term health impacts, such as response to antimalarial therapy, and long-term impacts, such as chronic anaemia and the impairment of cognitive development [5,6]. The burden of malaria can best be assessed using longitudinal studies with extended follow-up and comprehensive evaluation of outcomes. These studies also offer the opportunity to use a multi-disciplinary approach to explore these complex interactions [7]. Well-defined cohorts from the Gambia, Senegal, Kenya, Tanzania, and Mozambique have been instrumental in characterizing the epidemiology of malaria, exploring the acquisition of immunity, and demonstrating the effectiveness of control interventions such as insecticide-treated bednets, intermittent preventative treatment and vaccines [6,8-11].

Longitudinal studies of malaria have typically been conducted in rural communities with stable populations and relatively homogeneous transmission intensities and age-specific disease risk. However, sub-Saharan Africa currently has the highest rates of urbanization in the developing world [12]. These rapidly growing low-income urban communities differ from rural populations demographically, in socio-economic and cultural composition, and in access to treatment [13]. Urban malaria is usually defined by relatively low transmission intensity (entomologic inoculation rates less than 5 per person per year) with focal larval breeding sites, reduced mosquito dispersal and variable parasite prevalence rates [14]. A number of studies have demonstrated in densely-populated cities that malaria transmission, parasite prevalence, and malaria incidence may be centered in "micro-environments" [15-17]. Urban areas provide a unique opportunity for malaria control given existing infrastructure, technologic capabilities and greater access to high quality health services [18,19]. Quantitative assessment of the malaria burden in urban areas can help to specifically target integrated and cost-effective strategies to improve control and treatment [20].

A longitudinal cohort study in 300 children aged 6 months to 5 years followed for 1 year was previously completed in Kampala, Uganda. Malaria was common in this cohort, with over two treatments per person year in children under the age of five years. This study provided valuable data on malaria incidence and response to therapy, but was limited by a relatively small sample size and duration of follow-up, and the use of convenience sampling, which limited the ability to generalize results [21]. In July 2004, a second longitudinal cohort study designed to enroll, by probability sampling, 600 children between one and 10 years of age from a geographically defined urban slum of Kampala was initiated. Described below are the methods used to enroll this cohort and characterize the study area, census population and study participants.

## Methods

### Description of study site

Kampala is the capital of Uganda with an estimated 1.2 million persons living in 306,000 households according to a 2002 national census. Malaria is considered meso-endemic in this region, occurring throughout the year with peaks during rainy seasons from March-May and September-November.

The study was conducted in the Mulago III parish, which is a typical urban slum located near our study clinic at Mulago Hospital, Uganda's main tertiary referral center. The area is primarily residential, characterized by high population density with low-income single- or two-roomed housing units. The local economy is primarily dependent on petty commercial activities and small-scale farming. The parish has a large swamp area with poor drainage and frequent flooding during the rainy seasons. The principal mosquito vector is *Anopheles gambiae sensu stricto*. A study conducted in an adjacent parish showed 0.1 to 2.9 *A. gambiae *(11% infective) per house [22].

### Census and demographic survey

The goal was to recruit a representative cohort of 600 children aged 1–10 years to participate in a three-year longitudinal study to measure malaria incidence and compare the efficacy of three different combination therapies. To generate a sampling frame of households with appropriate aged children for recruitment and gather basic demographic information about the target population, a census and brief survey was conducted from July to October 2004. Prior to the start of the study, investigators met with elected government representatives to inform them of the census project and explain the methodology.

Over a four month period, teams of three study personnel systematically covered the entire area of Mulago III parish on foot to identify and enumerate all households. The households were without addresses and scattered along unnamed dirt roads and foot paths. A household was defined as any single permanent or semi-permanent structure acting as the primary residence for a person or group of people. Households were assigned sequential unique numbers, written on a visible label fixed above the doorway, and residents were given a household identification card. After enumeration, study personnel asked an adult resident (18 years of age or older) from each household for verbal consent to participate in a brief demographic survey. Using a standardized questionnaire, information was gathered on households, including construction materials and number of rooms, and the age, gender and bednet practices of each resident. A resident of a household was defined as a person who intended to sleep primarily at that location for the subsequent 6 months [23]. If no adult resident was available, households were revisited up to four times to complete the survey. The house was considered vacant if no residents were present at the household after four visits.

### Mapping the study area

To create a map of households and other points of interest, Mulago III Parish was charted using Pathfinder Pocket Global Positioning System (GPS) Receivers (Trimble Navigation Ltd., Sunnyvale, CA, USA) equipped with TerraSync 2.40 software (Trimble Navigation Ltd., Sunnyvale, CA, USA) connected to handheld personal digital assistants (PDA). A minimum of 12 satellite readings, with an accuracy goal of ~1 m, were taken from the door of each household. These values were average to obtain the final GPS reading (Easting, Northing, and Altitude) for each household in UTM units. Similar readings were taken for the boundaries of the parish and potential mosquito breeding sites such as swamps, cement drains, water channels, springs, closed water taps, and wells. A swamp was defined as a wet marsh-like field with papyrus plants. Large drains and water channels were defined as >1 meter wide. A protected spring was defined as a water source with a continuous flow of water through a pipe from a sheltered cement access point. A natural spring was defined as continuous flow of water from soil, rock, or pool. Closed water taps were defined as outdoor closed faucets with or without hose attachments, and wells were defined as large volume water storage containers. Additional readings were recorded for health care facilities (drug shops, pharmacies, public and private clinics, and hospitals) and other points of interest (schools, abattoirs). GPS data were transferred from the handheld PDA devices and synchronized with a Microsoft Access database using GPS Pathfinder Office version 2.90 (Trimble Navigation Ltd., Sunnyvale, CA, USA). Arc View version 3.3 (ESRI, Buckinghamshire, UK) was used to analyse geographical data and create a detailed map of the study area.

### Recruitment of the cohort

To recruit a representative cohort of children between 1–10 years of age for participation in the longitudinal study, probability sampling at the level of the household was used. Household level recruitment was chosen to meet the practical objective of enrolling all eligible children from each household and because residents, but not houses, may change between the census and recruitment periods. From a database of all households enumerated in the census, a random list of households with at least one child less than 10 years of age was generated. From November 2004 to April 2005, experienced home visitors approached households for recruitment sequentially from the randomized list, using handheld GPS receivers to locate households by previously recorded coordinates. Interviews were conducted to confirm that households had at least one child age 1 to 10 years. A brief description of the study was provided in the appropriate language to parents and/or guardians. For parent/guardian(s) interested in the study, an appointment was made for a screening interview at our study clinic. Residents not at home during the initial visit were revisited up to three times over the next six weeks to assess interest in the study.

### Screening and enrollment

Interested households were screened sequentially until 601 children were enrolled. All eligible children from each household screened were enrolled. Initial assessment of eligibility was conducted by study physicians for the following selection criteria; 1) age one to 10 years; 2) agreement to come to study clinic for any febrile episode or illness; 3) agreement to avoid medications administered outside the study; 4) agreement to remain in Kampala; 5) absence of known chronic disease or history of side effects to the study medications, and 6) written informed consent provided by parent or guardian. Children who passed initial screening were assigned study numbers and underwent a history and physical examination including temperature, height and weight. Children with weight < 10 kg, or severe malnutrition, as defined by weight-for-height or height-for-age Z-score < -3, were excluded. Blood was collected by venipuncture for thick blood smear, complete blood count, creatinine, alanine aminotransferase, bilirubin, haemoglobin electrophoresis, glucose-6-phosphate dehydrogenase (G6PD) activity, and storage for future molecular studies. Children returned to review results of screening labs, generally within 72 hours. Children who were homozygous for haemoglobin SS or had life-threatening screening laboratory abnormalities (based on toxicity grading scales developed by the World Health Organization and the National Institutes of Health) were excluded. Children with symptomatic malaria (fever and a positive thick blood smear) at the time of initial screening were treated with quinine (10 mg/kg tid × 7 days). Follow-up began when children fulfilled all of our selection criteria and were free of symptomatic malaria.

### Household survey

Within two weeks of enrollment, an appointment was scheduled for administration of a household survey to the primary caregiver for each enrolled household. The primary caregiver was defined as the person primarily responsible for daily care of the child. The questionnaire was administered at the home and collected basic demographic information about the study participant, primary care giver, and household, including use of prevention measures. Reported bednet use was verified by direct observation.

### Microscopy

Thick blood smears were stained with 2% Giemsa for 30 minutes. Parasite density was estimated by counting the number of asexual parasites per 200 white-blood cells and calculating parasites per μL, assuming a white blood cell count of 8,000 cells per μL. A smear was judged to be negative if no parasites were seen after review of 100 high-powered fields. A second microscopist, who was unaware of the results of the first reading, re-read all slides. A third reviewer resolved discordant results.

### Data management and analysis

Census data were collected in the field on written forms and transferred to an electronic data collection (EDC) system using Visual CE 8.1 software (Syware Inc, Cambridge, MA, USA) on a handheld PDA. The data were downloaded to Microsoft Access and then transferred into an Epi-Info version 6.04 database at the end of each day. Double entry verification from paper questionnaires was performed to assure accuracy. Given the success of the census EDC system, direct entry EDC questionnaire was used during the household survey, eliminating need for double entry verification. Categorical variables were compared using the chi-square test and continuous variables using a two-sample t-test. Independent predictors of anaemia and parasitaemia were identified using multivariate logistic regression. A p-value < 0.05 (two-tailed) was considered statistically significant. Analysis was done using SPSS version 10.0 (SPSS, Chicago, IL, USA) and Stata version 8.0 (Stata, College Station, TX, USA).

Verbal consent was obtained for the brief census survey described above. Written informed consent was obtained from the parent/guardian(s) of children for their participation in the cohort study and for the future use of biological specimens. The study was approved by the Institutional Review Boards at the University of California, San Francisco and Makerere University School of Medicine, and by the Ugandan National Council of Science and Technology.

## Results

### Census and mapping

**Figure 1:**
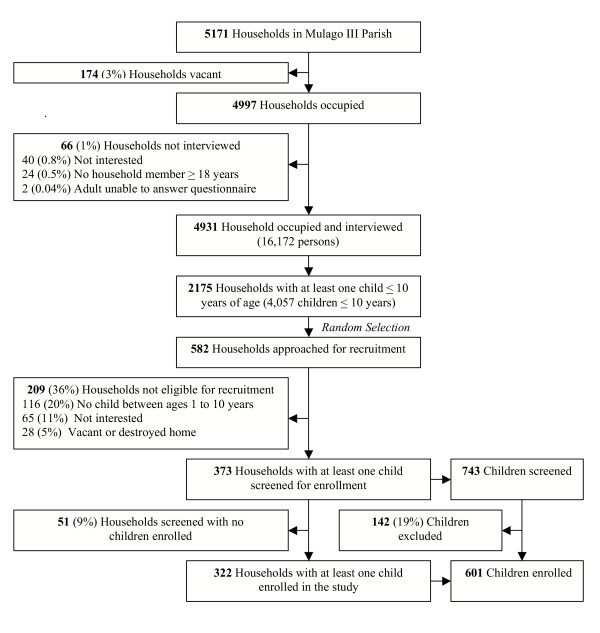
Schematic of census, household recruitment, and enrolment of children into longitudinal study. Reasons that children were excluded before enrolment: 42 (5.6%) did not give informed consent, 28 (3.8%) moving out of Kampala, 27 (3.7%) weight < 10 kg, 15 (2.0%) not willing to come to study clinic or avoid outside medications, 11 (1.5%) age not between 1 to 10 years, 7 (0.9%) history of serious chronic illness, 6 (0.8%) Z-score <-3SD for severe malnutrition, 3 (0.4%) sickle cell disease by reported history or homozygous haemoglobin SS by electrophoresis, 2 (0.3%) life threatening laboratory values and 1 (0.1%) history of side effects to study medications.

A total of 5,171 households were enumerated, of which 174 (3.4%) were vacant and 66 (1.3%) declined to consent for the demographic survey. The total census population was 16,172 people living in 4931 households (Figure 1). The parish covered an area of less than 1 square kilometer (0.86 km^2^), with an overall population density of 18,824 persons/km^2^. A majority of the homes were single rooms (68%) and the most common type of construction material was cement or concrete (80%). The mean number of persons per household was 3.3 (range 1–16) and the mean number of persons per room was 2.3 (range 0.2–11). A single adult aged 18 years or older inhabited 19% of the households and an additional 27% of households were inhabited by two or more adults without any children. The mean age of the census population was 21.2 years, with 25% less than 10 years of age and 13% less than 5 years of age (Table 1).

**Table 1:** Comparison of census population and enrolled households and children.

Characteristics of the household from census data	All Households (n = 4931)	Households with children aged 1 to 10 years not included in the study (n = 1672)	Households with children aged 1 to 10 years included in the study (n = 322)	P-value*
Median number of persons (range)	3 (1–16)	4 (2–16)	5 (2–16)	0.82
Median number of rooms (range)	1 (1–17)	1 (1–17)	1 (1–8)	0.24
Construction of home				
Cement or Concrete	78%	81%	80%	
Mud and wattle	16%	14%	14%	0.20
Mud or clay brick	6%	5%	5%	
Use of at least one bednet	54%	64%	63%	0.78
Use of at least one ITN	15%	18%	16%	0.36

Characteristics of the individual	Census Population (n = 16,172)	Children aged 1 to 10 years in census (n = 3590)	Children aged 1 to 10 years enrolled in study (n = 601)	P-value^†^

Mean age in years (SD)	21.2 (13.7)	5.3 (2.9)	5.8 (2.6)	<0.01
Gender (% female)	48%	51%	48%	0.12
Use of bednet	45%	48%	43%	0.03
Use of ITN	12%	13%	6%	<0.01

*Comparison of households with children aged 1 to 10 years not enrolled vs. enrolled households from the census data.

^†^Comparison of children aged 1 to 10 years enrolled interviewed at household survey vs. children aged 1 to 10 years from the census data.

**Figure 2:**
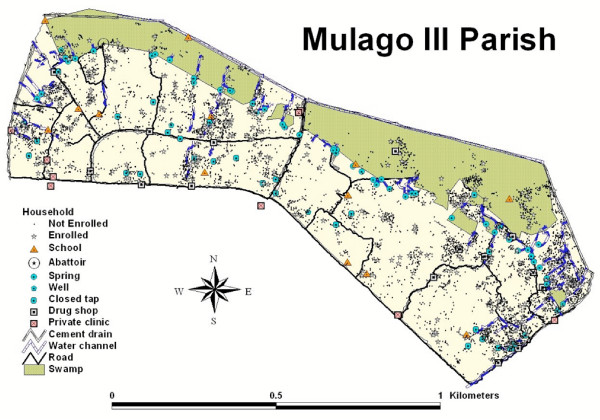
Map of the Mulago III parish study area in Kampala, Uganda.

Potential mosquito breeding sites within the parish included a large swamp area along the northern border (Figure 2). The majority of the 45 springs and water channels identified were along the southern border of the swamp. Twenty-three closed water taps were identified in the southern region of the parish. Cement drains carrying rain and waste water runoff were located along the main roads. Sixteen drug shops, 9 private health clinics, 11 primary and nursery schools, and 2 secondary schools were identified in the parish. The Mulago Hospital Complex, including our study clinic, is approximately 200 m from the parish. Households were concentrated along the main roads, markets and trading centers (Figure 2).

### Recruitment and enrollment of study participants

Of the 582 households that were approached for recruitment, 209 were excluded prior to screening for the following reasons: children aged 1 to 10 years no longer living in the household (20%), parents not interested in the study (11%), and homes vacated or destroyed since the time of census (5%). A total of 743 children from 373 households were screened in the clinic (Figure 1). The most common reasons for exclusion during screening included: parent/guardian unwilling to provide informed consent (6%), plans to move out of Kampala (4%), and weight < 10 kg (4%).

When the census database was analysed, there were no differences between households with children aged 1 to 10 years that were enrolled and those not enrolled in the study (Table 1). A similar proportion of enrolled households had at least one bednet when data collected during the household survey (using direct observation) was compared to data reported during the census (60% vs. 63%, P = 0.47). However, the proportion of enrolled households with at least one ITN that was directly observed during the household survey was lower than the proportion based on data reported during the census (10% vs. 16%, P < 0.01). Compared to children aged 1 – 10 years from the census, enrolled children were slightly older (5.8 years vs. 5.3 years, P < 0.01), probably because young children weighing less than 10 kg were excluded. Based on direct observation, enrolled children were less likely to use a bednet and less likely to use an ITN compared to data from children aged 1–10 years collected during the census (43% vs. 48%, P = 0.03 and 6% vs. 13%, P < 0.01, respectively).

### Baseline characteristics of study cohort

Characteristics of the children enrolled in the study are summarized in Table 2. A total of 81% of the enrolled children were born and raised in Kampala and 78% were under the care of their biological parents. Although children with severe malnutrition (Z-score < -3SD) were excluded, the majority of enrolled children had height-for-age and weight-for-height Z-scores below the median of the U.S. National Center for Health Statistics reference population. Few children were classified as stunted (8%), defined as height-for-age Z-scores < -2SD or wasted (2%), defined as weight-for-height Z-scores < -2SD. Haemoglobin electrophoresis revealed 99 (17%) children were heterozygous AS for sickle cell disease with no significant difference by gender (P = 0.43). Low G6PD activity was found in 80 (13%) of the enrolled children and males were more likely to have low G6PD activity compared to females (16% vs. 10%, P = 0.045). Mean haemoglobin (Hb) at enrollment was 11.8 g/dL (range 6.8–15.7 g/dL). A total of 24% of children were mildly anaemic (defined as Hb < 11 g/dL) and only 1% were moderately anaemic (defined as Hb < 8 g/dL). Multivariate analysis revealed increasing age (OR = 0.62 for each 1 year increase in age, 95% CI 0.56–0.68, P < 0.001) and bednet use (OR = 0.58, 95% CI 0.38–0.91, P = 0.02) were associated with a decreased risk of mild anaemia. Gender, G6PD activity, and sickle cell trait were not associated with mild anaemia. At enrollment, 19% children were parasitaemic (geometric mean parasite density = 1210 parasites/μL, range 16 – 320,800/μL) and 3% had symptomatic malaria (defined as fever and parasitaemia). Multivariate analysis revealed that low G6PD activity (OR = 0.33, 95% CI 0.15–0.77, P = 0.009) and bednet use (OR = 0.64, 95% CI 0.41–0.99, P = 0.045) were associated with a decreased risk of parasitaemia. Increasing age (OR = 1.08 for each 1 year increase in age, 95% CI 1.00–1.17) was associated with an increased risk of parasitaemia, but did not reach statistical significance (P = 0.06). Gender and sickle cell trait were not associated with parasitaemia. Children who were parasitaemic at enrollment were much more likely to also be mildly anaemic (OR = 3.89, 95% CI 2.28–6.65, P < 0.001) after controlling for age, sickle cell trait, low G6PD activity and bednet use.

**Table 2:** Baseline characteristics of study cohort (n = 601).

Characteristic	Summary (No. (%))
Relationship to primary caregiver	
Child (Mother)	406 (68%)
Child (Father)	59 (10%)
Niece/Nephew	60 (10%)
Grandchild	52 (9%)
Born and raised in Kampala	484 (81%)
Anthropometric indices^†^	
Mean height for age Z-score (SD, range)	-0.63 (1.2, -2.9 – 6.1)
Mean weight for height Z-score (SD, range)	-0.29 (0.9, -2.8 – 5.3)
Height for Age < -2SD	48 (8%)
Weight for Height < -2SD	10 (2%)
Haemoglobin electrophoresis	
AA	502 (84%)
AS	99 (17%)
Low G6PD activity*	
Males	50 (16%)
Females	30 (10%)
Mean haemoglobin at enrollment (SD, range)	11.8 (1.4, 6.8–15.7)
Anaemia (Hb<11 g/dl)	144 (24%)
Moderate anaemia (Hb<8 g/dl)	6 (1%)
Asymptomatic parasitaemia	101 (17%)
Symptomatic malaria^§^	15 (3%)
Gametocytes present	43 (7%)

^†^Children < 8 years (n = 446)

*Less than 118 mU/10^9 erythrocytes per manufacturer

^§^Symptomatic malaria defined as positive blood smear and temperature ≥38.0°C or history of fever in last 24 hours.

### Use of bednets

**Figure 3:**
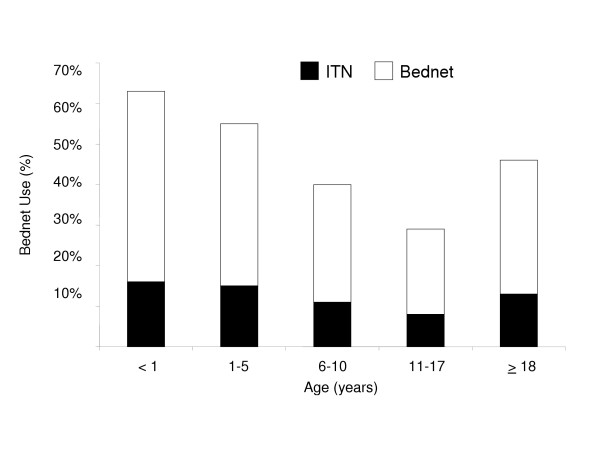
Bednet and insecticide-treated net use by age category in the Mulago III parish census population. For all pairwise comparisons, P < 0.01.

Among the census population, bednet use was reported by 45% with 12% reporting use of an ITN. Bednet coverage was highest among children less than one year of age (63%) and decreased with increasing age through adolescence (29% in persons aged 11 to 17 years) followed by an increase in adults 18 years or older (46%) (Figure 3). Trends in ITN use across age groups were similar; however, only 27% of bednets were reported to be treated with insecticide. Among persons using a bednet, 89% reported sleeping under the net "always", 10% reported "sometimes" and 2% reported "rarely". There was no statistically significant difference in bednet use by gender among children less than 18 years of age (P = 0.09). Among adults, females were significantly more likely than males to report use of any bednet or an ITN (54% vs. 41%, P < 0.0001 and 16% vs. 11%, P < 0.0001, respectively).

Additional details about bednet use were collected during the household survey for enrolled children. Only 6% of enrolled children used an ITN and 68% of these ITNs were either new or retreated within the last 6 months. Observed bednets were generally in poor condition with 56% having visible holes.

## Discussion

Longitudinal studies in well-defined cohorts are critical to improving our understanding of the complex epidemiology of malaria in urban areas. In Africa, longitudinal studies of malaria have traditionally been conducted in rural villages where stable populations are well-suited to participate. However, malaria risk in urban environments has been recognized as a growing problem, and data from rural settings may not be applicable to urban settings. It has been estimated that half of Africa's population will live in urban areas by 2025 [12]. Compared to rural areas, intense focal transmission near urban breeding sites leads to clustering of malaria incidence, resulting in a smaller proportion of households carrying most of the malaria burden [16,18]. In addition, urban populations often demonstrate a delayed acquisition of immunity which could lead to a higher risk of disease in older age groups [24]. The lack of longitudinal studies of malaria control in urban areas may be due to lower disease incidence, the effort required to sample from rapidly growing and densely populated communities, and the transient nature of urban residents. In this study, the methodology used to enroll a representative cohort of children from an urban environment was described and some of the advantages of this type of study are highlighted, together with the difficulties encountered.

By enumerating a census population from a geographically defined residential area near the study clinic, it was possible to characterize a target population and generate a sampling frame for recruitment of a representative cohort of children. The census depicted a community with high population density (nearly double that of New York City) and high household occupancy, where the majority of homes were permanent, cement, single-room structures. The difficulty of enumerating homes without addresses densely concentrated along unpaved roads and foot paths was overcome by labeling homes with identification numbers and recording GPS coordinates. Eliciting local elected officials to participate as part of the census contributed to the success of the project due to their familiarity with the community. One obstacle encountered was that adult residents were frequently not home during daytime hours, reflective of a working class adult population.

Compared to rural areas, where households are typically inhabited by large extended families, the census population had smaller household sizes with over 40% inhabited by a single adult resident or one or more adult residents without children [25]. Only 40% of the census population was under 18 years of age, compared to nearly 60% in rural Uganda [26]. In this census, 45% of households reported owning at least one bednet, which was higher than urban areas (33%) and considerably higher than rural areas (9%) previously reported in Uganda [26]. Bednet use was highest in the youngest age groups, declined during adolescence and then increased in the adult population over the age of 18 years, when females were significantly more likely than males to use a bednet.

Probability sampling of households enumerated during the census was used to recruit a cohort of 601 children one to 10 years of age for our longitudinal study. Only 44% of households enumerated in the census had at least one child one to 10 years of age and were eligible for recruitment. This age range was chosen because one of the study regimens (artemether-lumefantrine) was not approved for use in children less than 10 kg when the study was designed. In addition, the relatively delayed development of acquired malaria-specific immunity in low endemicity areas like urban Kampala puts older children at risk. Recruitment was conducted at the household level because households enumerated during the census would likely remain constant, although residents may change. Only 45% of the households approached contributed at least one child to the study. Therefore, using random selection at the household-level, it was necessary to approach nearly double the number of households needed to enroll the proposed sample of 600 children. This was primarily due to children no longer residing in the household when it was approached for recruitment, likely secondary to the common practice of sending children to less expensive boarding schools outside of Kampala. The efficiency of the recruitment process was also reduced by a large proportion of households with future intentions to move from Kampala and a considerable proportion of households that were uninterested in the study, often stating that the offer of free health care was not an incentive given their access to health insurance. GPS technology afforded a significant advantage, enabling home visitors to relocate eligible households in the densely populated community from previously recorded coordinates. Experience with the census and recruitment process in this study population suggests that a broad study area and sizeable recruitment strategy is needed to carry out population-based random sampling for paediatric clinical studies in urban areas.

Cross-sectional analysis of anaemia and parasitaemia in the enrolled cohort of children revealed some risk factors for malaria in this community. The prevalence of mild anaemia was low (24%) and, consistent with prior studies, significantly more common in younger children and less common in children using bednets [27]. Mild anaemia in children in the cohort between one to five years of age (44%) was lower than that reported from national surveillance data for children of that age group in urban and rural areas of Uganda (51% and 67%, respectively) [26]. Malaria endemicity is classically defined by surveys of parasite prevalence. The Mulago III parish would be considered meso-endemic, defined as parasite prevalence of 11–50% in children of two to nine years of age, given a parasite prevalence of 20% in this age group. Increasing age showed a non-significant trend for an increased risk of parasitaemia, but low G6PD activity and use of bednets appeared to be protective. In addition, children with parasitaemia were significantly more likely to have mild anaemia. These predictors of anaemia and parasitaemia identified by cross-sectional analysis are applicable to other meso-endemic urban African environments.

Given the feasibility of deployment and the impact of ITNs on reducing malaria transmission and child mortality, high coverage rates should be sought in urban areas [28]. Use of any bednet by enrolled children less than five years of age (57%) was higher than previously reported in urban (21–34%) and considerably higher than rural areas (6–18%) in Uganda [29]. In addition, ITN use in enrolled children less than five years of age (15%) was higher than ITN use reported in urban areas (1–6%) and rural areas (0.2–2%) in Uganda [29]. It was also found that census surveys based on subjective reporting may overestimate the actual use of bednets given the significantly lower use of bednets and ITNs noted when nets were directly observed for the enrolled children during the household survey. Although measures of socioeconomic status were not controlled, cross-sectional analysis in this cohort suggests that use of bednets lowers the risk of parasitaemia and anaemia, reinforcing the well-known benefit of bednet use in children. To improve malaria control, increased ITN use was prioritized by African leaders at the Roll Back Malaria Summit in Abuja with a goal of 60% of those at risk of malaria, especially children under five years of age and pregnant women, to have access to ITNs by 2005. Despite the high level of bednet use in the census population, the use of ITNs in this community still remains far below the Abuja target.

The study site for this project has extensive resources to conduct a longitudinal clinical trial. Mulago Hospital complex is a tertiary care hospital open 7 days a week, including a paediatric acute care unit open 24 hours a day. On-site laboratory testing and comprehensive health services staffed by qualified physicians and scientists, provides access to prompt diagnosis and strict quality control standards. Future multidisciplinary investigations within the longitudinal study to identify predictors of malaria incidence and response to therapy include the impact of environmental risk factors, household characteristics and measures of socioeconomic status, and molecular studies investigating the role of immune responses and genetic polymorphisms in the host and parasite. In addition, probability sampling from the census used for enrolling the representative cohort of children will allow generalization of these results to growing urban populations.

## Conclusion

The challenge of decreasing the malaria burden in endemic areas is rooted in the complex epidemiology of this disease. Due to underlying differences between rural and urban areas, prevention and treatment strategies may need to be adapted to urban settings [23]. Fortunately, with the existing infrastructure available in most cities and the focal nature of transmission, urban malaria may be uniquely amenable to control interventions. For example, greater access to formal health care facilities may allow for more accurate diagnosis and appropriate therapy, while high population densities may facilitate increased bednet coverage [19]. Performing a census in a defined study area allows researchers to characterize their target population and generate a sampling frame for recruitment of representative cohorts. Such cohorts are critical for undertaking more comprehensive longitudinal studies in urban areas to identify environmental, household and individual-level predictors of malaria incidence and help to target control strategies aimed at reducing malaria morbidity and mortality in this increasingly important population.

## Authors' contributions

JD, SK, SS, and DG conceived and designed the study. JD, TC, SK, NT, and DN participated in data collection. JD, TC, SK, and GD participated in the data analysis. All authors were participated in the writing of the manuscript.

## Financial support

Financial support was provided from the National Institutes of Allergy and Infectious Disease (AI052142) and Fogarty International Center/National Institutes of Health (TW00007)
